# Oxidative stress and antioxidants in the trabecular meshwork

**DOI:** 10.7717/peerj.8121

**Published:** 2019-11-26

**Authors:** Mingxuan Wang, Yajuan Zheng

**Affiliations:** Department of Ophthalmology, 2nd hospital affiliated to Jilin University, Changchun, China

**Keywords:** Trabecular meshwork, Oxidative stress, Antioxidants, POAG

## Abstract

Glaucoma is an age-dependent disease closely related to oxidative stress and is regarded as the second leading cause of irreversible blindness worldwide. In recent years, many studies have shown that morphological and functional abnormalities of the trabecular meshwork (TM) are closely related to glaucoma, especially with respect to oxidative stress. In this review, the mechanisms of oxidative stress in the TM and treatment strategies for this condition, including strategies involving antioxidants, noncoding RNAs and exogenous compounds, are discussed. Although many questions remain to be answered, the reviewed findings provide insights for further research on oxidative stress alleviation in glaucoma and suggest new targets for glaucoma prevention.

## Introduction

**Figure 1:**
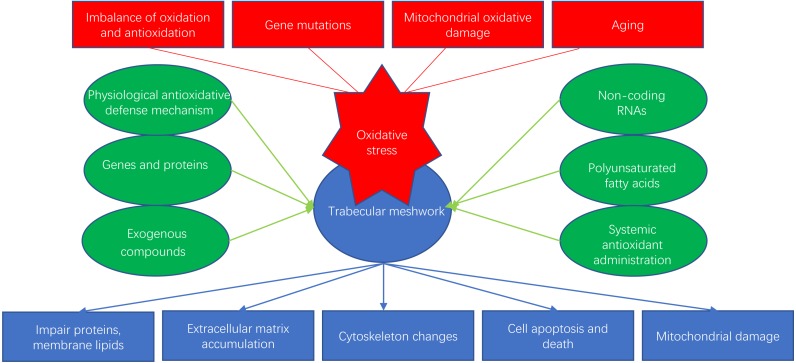
Oxidative stress and antioxidants of trabecular meshwork.

Glaucoma is an age-dependent disease closely related to oxidative stress and is considered to be the second leading cause of irreversible human blindness worldwide, especially in the elderly population (Quigley & Broman, 2006). Oxidative stress can happen in many ocular cells, such as corneal epithelial cells (CECs), trabecular meshwork (TM) cells (TMCs), retinal pigment epithelial cells (RPEs) and retinal ganglion cells (RGCs). In particular, oxidative stress-induced dysfunction of TMCs can obstruct the outflow of the aqueous humor, leading to pathologically high intraocular pressure (IOP) and contributing to glaucoma. Several studies have suggested that the progression of primary open-angle glaucoma (POAG) may be related to reductions in the antioxidant capacity of the TM (Ammar, Hamweyah & Kahook, 2012a). In this review, we discuss the mechanisms of oxidative stress and recent research on antioxidative strategies for the TM (Fig. 1).

## Survey Methodology

This review focuses on hot topics in glaucoma research: oxidative stress and antioxidants. All references were retrieved using search engines such as PubMed and Web of Science using keywords including “trabecular meshwork cells”, “oxidative stress”, “antioxidants” and “glaucoma.”

### ROS and oxidative stress

Free radicals are substances with unpaired electrons that are regularly produced through normal metabolic processes. Free radicals can be divided into oxygen and nonoxygen radicals, although oxygen free radicals account for 95% of all free radicals (Zhao et al., 2016). Oxygen radicals include oxygen and highly reactive oxygen molecules, such as hydrogen peroxide (H_2_O_2_), hydroxyl radicals (OH•), peroxide hydroxyl radicals, alkoxy radicals, superoxide and anionic radicals (O_2_-), which are collectively referred to as reactive oxygen species (ROS). The nicotinamide adenine dinucleotide phosphate (NADPH) oxidase family is an enzyme family whose main function is to produce ROS upon stimulation by different growth factors and cytokines in various cell types (Wulf, 2002; Yun et al., 2011). Important exogenous stimulants of free radical production include electromagnetic radiation (visible, ultraviolet (UV), and infrared radiation) and known environmental pollutants such as tobacco smoke. Endogenous sources of free radicals include mitochondria, which form superoxide through the respiratory chain, and polynuclear cells in the inflammatory environment, which perform important functions during the physiological response to injury.

Oxidative stress is usually caused by imbalance between ROS production and elimination as a result of biological defense mechanisms, mitochondrial dysfunction, impaired antioxidant systems or a combination of these factors. Oxidative stress increases the production of ROS, creating a vicious cycle. Abnormal ROS accumulation can cause oxidative damage to deoxyribonucleic acid (DNA), proteins, and lipids. DNA damage can induce apoptosis, autophagy, and mutation, which are associated with cataracts, age-related macular degeneration (AMD), retinopathies, and glaucoma.

### TM oxidative stress and glaucoma

Patients with POAG are susceptible to oxidative damage because their total reactive antioxidant capacity is 60%–70% lower than that of healthy individuals (Ferreira et al., 2004; Tanito et al., 2015). POAG patients’ serum samples always exhibit low levels of circulating glutathione (Doina et al., 2005), total antioxidant capacity (TAC), advanced oxidation protein products (AOPPs), superoxide dismutase (SOD), glutathione peroxidase (Gpx) (Engin et al., 2010) and catalase (CAT) (Majstereka et al., 2011) but high levels of malondialdehyde (MDA). Interestingly, results obtained from serum samples are consistent with those obtained from aqueous humor samples (Nucci et al., 2013; Sorkhabi et al., 2011), indicating that systemic antioxidant capacity can reflect local ocular redox status. Various studies have shown that TMCs are some of the most ROS-sensitive cells in the anterior chamber (Alberto et al., 2009) and serve as regulators of aqueous humor outflow. The TM structure can sustain oxidative stress due to the effects of UV-based oxidative byproducts of aqueous, corneal and crystalline epithelial cells (Stamer & Clark, 2017). ROS-mediated damage to the TM has been shown to impair the structural and functional components of mtDNA in TMCs and to damage proteins and membrane lipids (Abu-Amero, Jose & Bosley, 2006), increasing aqueous humor outflow resistance (Izzotti et al., 2003). Furthermore, elevations in IOP may accelerate oxidative adduct formation, which is greatest near neuronal cell bodies, resulting in a positive feedback loop (Weinreb & Tee, 2004). In vitro studies have shown that oxidative stress is often induced by hydrogen peroxide at different concentrations (Ammar, Hamweyah & Kahook, 2012b; Liu & Zhang, 2019; Lu & Wang, 2017; Zhao et al., 2019a) or by homocysteine (You et al., 2018) and rotenone (He et al., 2019). In addition, oxygen free radical generation in TMCs may increase with age, leading to gradual increases in oxidative damage, extracellular matrix (ECM) accumulation, cytoskeletal changes, apoptosis and changes in the structures and functions of plasmids and lysosomes (Gabelt & Kaufman, 2005).

**(1) Imbalance between oxidation and antioxidation in the anterior chamber**

Imbalance between oxidants and antioxidants or excessive ROS accumulation can cause oxidative stress (Aydin Yaz et al., 2019). Under conditions of oxidative stress, TMCs express a variety of reductases, such as SOD, glutathione S-transferase (GS-T), and GPx, that neutralize the active substances, and total antioxidant status (TAS) (Abu-Amero et al., 2011), CAT, vitamin C (Ferreira & Lerner, 2008), paraoxonase, and arylesterase can be measured as antioxidant markers. Furthermore, total oxidative stress (TOS) (Dursun et al., 2015), MDA (D’Azy et al., 2016) (16, 17), 8-hydroxydeoxyguanosine (8-OHdG) (Sorkhabi et al., 2011), 4-hydroxynonenal (4-HNE), protein carbonyl (PC) (Mesut et al., 2011), and nitric oxide (NO) have been measured as pro-oxidant markers in various studies. Other inflammatory markers, such as interleukin-1 α (IL-1 α) and endothelial leukocyte adhesion molecule (ELAM)-1, have been evaluated in animals and TMCs (Avotri, Eatman & Russell-Randall, 2019). Imbalance between oxidants and antioxidants can lead to ROS accumulation, TMC structural remodeling, TM enlargement or TM collapse. In addition, oxidative stress stimulates the migration of human TMCs in vitro, resulting in thickening, enlargement and fusion of the TM (Hogg et al., 2000)

**(2) Genes and mutations**

CyP1B1

Cytochrome P450 family 1 subfamily B member 1 (CYP1B1) is part of the CYP450 family, whose main function is to catalyze reactions of exogenous and endogenous molecules through NADPH (Savas et al., 1994). Mutations in CYP1B1 have been found in patients with congenital glaucoma. Appropriate expression of periostin (Postn) helps to maintain the structural integrity of TM tissue, and the expression of this molecule is influenced by Cyp1b1 (Yun et al., 2013)

LTBP2 (https://www.ncbi.nlm.nih.gov/gene/4053)

The latent transforming growth factor (TGF)- β binding protein (LTBP) 2 gene encodes the protein LTBP2, which is closely connected with ECM molecules including fibrillin proteins and other LTBPs (Rifkin, 2005). Knockdown of LTBP2 affects not only the ECM but also TMC apoptosis through a mechanism that may be mediated by the TGF β and BMP signaling pathways; these effects are similar to those induced by oxidative stress (Suri, Yazdani & Elahi, 2018).

MYOC

Myocilin (MYOC) is the first gene whose mutations were demonstrated to cause familial forms of glaucoma (Stone et al., 1997). One mutation in MYOC activates the IL-1/NF- κB pathway, significantly stimulating IL1A and IL1B expression, which may be associated with POAG (Itakura, Peters & Fini, 2015).

8-OHdG

8-OHdG, a product of oxidative damage to DNA, is produced by reaction of hydroxyl radicals with deoxyguanosine, which causes c-8-hydroxylation (Sun, 2016). As an endogenous mutagenic agent, 8-OHdG can cause a G:C → T:A mutation. One study using 8-OHdG as a marker of oxidative stress revealed that oxidative DNA damage is significantly elevated in TMCs of patients with POAG compared to TMCs of healthy individuals (Sacca et al., 2005). Further analysis revealed a significant positive correlation of 8-OHdG levels in the TM with visual field defects and increased IOP (Sergio Claudio et al., 2005).

TXNRD2

The thioredoxin reductase 2 (TXNRD2) gene encodes a mitochondrial protein of the same name that belongs to the pyridine nucleotide-disulfide oxidoreductase family and is a member of the Trx system. This protein is necessary for reducing damaging ROS generated by oxidative phosphorylation (OXPHOS) and other mitochondrial functions (Chen, Cai & Jones, 2006). A genome-wide association analysis reported that TXNRD2 loci are significantly associated with POAG (Shiga et al., 2018). Additionally, Bailey et al revealed that TXNRD2 loci are significantly associated with IOP in another genome-wide association study (Bailey et al., 2016).

**(3) Humor outflow impairment and the ECM**

Excessive accumulation of ECM proteins (e.g., collagen, fibronectin (FN), and laminin) in the TM may induce elevations in IOP. In vitro induction of oxidative stress in TMCs leads to typical POAG-like changes (ECM accumulation, cell death, cytoskeletal disorders, inflammatory marker release, etc.), which can be significantly reduced by pretreatment with antioxidants and vasopressors (prostaglandin analogs and carbonic anhydride inhibitors) (Welge-Lussen & Birke, 2010). The levels of FN, an ECM component, are significantly increased in the context of POAG. Increased FN concentrations can not only cause TMC dysfunction but also reduce the numbers of TMCs, thus affecting normal aqueous filtration (Hogg et al., 2000). FN can also change the structures of TMCs, causing dysfunction (Padma et al., 2012). In addition, FN can change other ECM characteristics, increasing the outflow resistance of the aqueous humor. With regard to DNA damage, continuous oxidative stress decreases the function of miR-29b, which negatively regulates the expression of ECM-related genes, thereby promoting the deposition of ECM in the TM and impeding the flow of water out of the chamber (Luna et al., 2009).

**(4) Mitochondrial oxidative damage in TMCs**

Mitochondria are important sites of intracellular aerobic respiration that play vital roles in maintaining cell homeostasis by regulating processes including oxidative energy metabolism, intracellular calcium balance, neuronal excitability and synaptic transmission, and apoptosis (Chan, 2006). Mitochondrial dysfunction can decrease intracellular ATP synthesis and inhibit mitochondrial OXPHOS, inducing excessive ROS production. Excessive accumulation of ROS leads to mitochondrial DNA damage, which further damages mitochondrial structure and function and in turn generates additional ROS. In recent years, increasing evidence has shown that mitochondrial injury and oxidative stress are involved in TMC damage in glaucoma (Zhao et al., 2016). Mitochondrial complex I defects have been reported to be associated with the degradation of TMCs in POAG patients (Yuan et al., 2008). In addition, patients with POAG are more likely to have a maternal family history than a paternal family history, suggesting a role for mitochondrial inheritance (Paul et al., 2002). Abu-Amero et al. (2011) found 27 nonsynonymous mtDNA mutations in POAG patients, 22 of which were potentially pathogenic, while no such mutations were found in a healthy control group. Mean mitochondrial respiratory activity was decreased in 24 cases, further indicating that oxidative stress and mitochondrial dysfunction contribute significantly to POAG. Chen, Cai & Jones (2006) found that the redox status of mitochondrial thioredoxin (mtTrx) underlies the vulnerability of mitochondria to oxidative injury. These findings indicate that glaucoma is a mitochondrial neurodegenerative disease and thus may suggest new options for glaucoma treatment.

**(5) Inflammatory response to oxidative stress**

Previous results (Li et al., 2007a) have revealed that the pathological changes induced by oxidative stress include cell death, intracellular ROS production, proinflammatory factor induction, senescence marker activation, PC accumulation, proteasome activity promotion, and apoptosis promotion, all of which are hallmarks of glaucoma. Inflammatory cells release active substances at inflammatory sites, leading to excessive oxidative stress (Li et al., 2007b). Reactive oxygen and nitrogen species (RONS) can activate the expression of proinflammatory genes through intracellular signaling cascades (Yang et al., 2012). For example, ROS can activate the NF-κB pathway, whose downstream target genes include components of mitogen-activated protein kinase (MAPK) signaling pathways, phosphoinositide 3-kinase (PI3K)-Akt, extracellular signal-regulated kinase (ERK) and p38 (Li et al., 2007b), which may alter TM mobility and cause contractile dysfunction. Additionally, oxidative stress can increase the expression of some inflammatory mediators, including IL-1α, IL-6, IL-8 and ELAM-1, not only in glaucomatous TMCs but also in vivo (Tourtas et al., 2012). This effect is further exacerbated by upregulation of the expression of ELAMs due to oxidative stress and activation of the inflammatory cytokine IL-1. Sirtuin 1 (SIRT1) is a member of the sirtuin family of nicotinamide adenine dinucleotide (NAD+)-dependent histone deacetylases; this protein helps to regulate lifespan in several organisms and may provide protection against diseases related to oxidative stress-induced ocular damage. In the case of glaucoma, such protection is likely to occur through the interaction of SIRT1 with endothelial nitric oxide synthase (eNOS) (Thomas et al., 2002), which regulates inflow and outflow pathways of TMCs.

**(6) Aging and oxidative stress**

Aging refers to the gradual loss of tissue and organ functions over time (Losordo & Henry, 2016). Aging, in which oxidative stress plays a major role, is a risk factor frequently associated with various degenerative diseases. Age-related structural damage and functional loss are due to the accumulation of oxidative damage in macromolecules (lipids, DNA and proteins) mediated by electrons (Beckman & Ames, 1998). The TM shows striking morphological decay during aging; its cellularity diminishes in a linear manner with age. The exact mechanism by which oxidative stress induces senescence is unclear, but increased RONS levels are known to cause cellular senescence. Autophagy plays a critical role in the removal of aged or damaged intracellular organelles and in the delivery of damaged organelles to lysosomes for degradation (Cuervo et al., 2005). Aging promotes TM senescence due to increased oxidative stress, and this process is paralleled by increased autophagy (Pulliero et al., 2014). Furthermore, production of advanced glycation end products (AGEs) is induced by nonenzymatic reactions between sugars and proteins under conditions of abnormally increased glucose concentrations, especially in aged patients or in patients with diabetes mellitus (Bucala, Tracey & Cerami, 1991); AGEs can enhance TMC senescence and increase oxidative stress (Park & Kim, 2012).

### Antioxidative strategies

** (1) Physiological antioxidative defense mechanisms**

Physiological antioxidative defense mechanisms involve a number of enzymes, such as SOD, CAT, GPx, GS-T, and the thioredoxin (TRX) system (Rokicki et al., 2016). Nonenzymatic antioxidants include endogenously produced GSH and dietary compounds, such as vitamins C and E (Zanon-Moreno et al., 2013); vitamin-like antioxidant compounds, including polyphenols and oligoelements; and certain metalloreductases. The function of these antioxidants is to capture free radicals by accepting and transferring unpaired electrons or through UV light absorption. In addition to the antioxidants described above, TMCs have been shown to be able to synthesize β-crystalline as a molecular chaperone to prevent oxidative damage (Pinazo-Durãn et al., 2017).

** (2) Genes and proteins**

FOXC1

Forkhead box C1 (FOXC1) is a member of the Forkhead Box or FOX class of transcription factors. The FOX class regulates cellular functions, the development of many organ systems, energy homeostasis and oncogenesis (Carlsson & Mahlapuu, 2002; Lehmann et al., 2003). FOXC1 is essential for the survival of TMCs under conditions of oxidative stress (Berry et al., 2008).

Prdx6

Peroxiredoxin 6 (Prdx6), a protective protein together with GPx and acidic calcium-independent phospholipase A2, acts as a rheostat to regulate cellular physiology by clearing ROS (Singh et al., 2016). ROS accumulation and pathobiological changes in aging or glaucomatous TMCs are partly due to the loss of Prdx6 (Chhunchha et al., 2017) and are correlated with increases in senescence markers and reductions in telomerase activity.

HES1

Hairy and enhancer of split 1 (HES1), which belongs to the basic helix-loop-helix family of transcription factors, is a transcriptional repressor. HES1 regulates the development of cells in the nervous and digestive systems by functioning downstream of the Notch signaling pathway (Kageyama, Ohtsuka & Kobayashi, 2007). Xu et al. found that HES1 promotes ECM expression and inhibits TMC proliferation and migration under oxidative stress (Xu et al., 2017). More importantly, HES1 short hairpin RNA (shRNA) has been shown to attenuate ECM protein upregulation and functional defects caused by oxidative stress.

TGF-β2

TGF-β2 in the aqueous humor may cause molecular changes and increase outflow resistance in POAG (Inatani et al., 2001; Junglas et al., 2009). The effect of connective tissue growth factor (CTGF) in oxidative stress is associated with ECM synthesis and increased contractility of the TM, contributing to a decrease in aqueous humor outflow facility and an increase in IOP (Sabrina et al., 2015). A recent study showed that mitochondrial-targeted antioxidants (XJB-5-131 and MitoQ) can attenuate TGF- β2/Smad signaling in TMCs through processes including reductions in CTGF and collagen isoform gene and protein expression (Rao et al., 2019).

NRF2

Nuclear factor (erythroid-derived 2)-like 2 (NRF2) plays a key role in regulating cellular oxidation reactions through oxidative stress defense mechanisms (Sachdeva, Cano & Handa, 2014). After exposure to ROS, Kelch-like ECH-associated protein 1 (Keap1) undergoes conformational changes, translocating NRF2 into the nucleus, binding to the antioxidant response element (ARE) region, and initiating the transcription of targets, including heme oxygenase-1 (HO-1) (Batliwala et al., 2017; Suzuki & Yamamoto, 2015) and NAD (P)H:quinone oxidoreductase1 (NQO1). Recently, many NRF2 activators, including the antioxidants sulforaphane (SFN), quercetin, and resveratrol (RSV), have been intensively studied and show great potential for protection against oxidative stress; these findings may offer new strategies for glaucoma treatment.

Rho kinase family members and their inhibitors

The Rho family kinases (Pinazo-Durãn et al., 2017) and their inhibitors (AMA0076, AR-13324, K-115, PG324, Y-39983, RKI-983, H-1152 recoverin and Y-27632) (Fujimoto et al., 2017) modulate signal transduction pathways; actin cytoskeleton function; and TMC, canal of Schlemm and ciliary muscle cell motility. In vivo, inhibition of p38 MAPK phosphorylation decreases tert-butyl hydroperoxide-induced apoptosis in TMCs.

** (3) Noncoding RNAs**

MicroRNAs are a class of small noncoding RNAs (19–25 nucleotides in length) that regulate a wide range of cellular processes by repressing the transcription or translation of their target genes (Van Rooij, 2011). MiRNAs are abundantly present in biological fluids and are reliable diagnostic and predictive biomarkers (Weber et al., 2010). Long noncoding RNAs are >200 nucleotide-long RNA molecules that lack or have limited protein-coding potential but can regulate miRNAs or protein formation through several different mechanisms (Wawrzyniak et al., 2018). Recently, noncoding RNAs have become popular subjects of glaucoma research (Table 1), providing attractive opportunities to defend against oxidative stress and to identify novel biomarkers for the diagnosis and prognosis of glaucoma.

**Table 1:** Role of antioxidative stress of miRNAs and lncRNAs.

Name	Functions and Mechanisms	References
miR-1	Regulates TMCs under oxidative stress by targeting FN expression.	Guo et al. (2019)
miR-29b	Downregulated by TGF-β_2_ and oxidative stress. Negatively regulates the expression of multiple genes involved in the synthesis and deposition of ECM proteins, including SPARC (secreted protein, acidic, and rich in cysteine), FBN1, laminin, collagens, BMP1, ADAM12, NKIRAS2, and SP1.	Guadalupe et al. (2011), Li et al. (2009), Luna et al. (2009), Srikumar et al. (2008), Zhaoyong et al. (2009)
miR-21	Increases the production of the ECM by silencing its target gene PTEN and by regulating TGF-β_2_ expression.	Dang (2017)
miR-181a	Inhibits the TMCs apoptosis induced by H_2_O_2_ through the suppression of the NF-*κ*B and JNK pathways.	Wang et al. (2018)
miR-1298	Protects TMCs against the damage caused by chronic oxidative stress (COS) via inhibiting the TGF- β_2_/Smad4 pathway and activating the canonical Wnt pathway.	Ruibin et al. (2018)
miR-483-3p	Inhibits the ECM after oxidative stress by targeting Smad4.	Shen et al. (2015)
miR-24	Regulates TGF *β*_1_ during cyclic mechanical stress by targeting FURIN.	Coralia et al. (2011)
miR-200c	Inhibits the expression of genes (ZEB1, ZEB2, FHOD1, LPAR1/EDG2, ETAR, and RHOA) related to the contraction of TMCs.	Luna et al. (2012)
miR-146a	Modulates inflammatory markers.	Guorong et al. (2010)
miR-204	Affects the sensitivity of TMCs to apoptosis and the number of cells. Acts as a direct target of AP1S2, Bcl2l2, BIRC2, EDEM1, EZR, FZD1, M6PR, RAB22A, RAB40B, SERP1, TCF12, TCF4, CLOCK, PLEKHG5, and ITGB1 MEIS2 and as a potential target of FOXC1.	Guorong et al. (2011), Matthew et al. (2012), Paylakhi et al. (2013), Redis et al. (2012)
miR-155	Regulates the ECM though interacting with the TGF β pathway.	Bjoern et al. (2006), Johannes et al. (2004)
miR-184	Regulates the growth, apoptosis and cytotoxicity by inhibiting HIF-l α.	Wang et al. (2017)
miR-93	Inhibits the viability and induces the apoptosis via the suppression of NRF2.	Wang, Li & Wang (2016)
miR-175p	MiR-17-5p was downregulated in TMCs under oxidative conditions, and may regulate the apoptosis of TMCs by targeting PTEN	Wang et al. (2019)
miR-27a	Regulates Nrf_2_ expression at the posttranscriptional level. Salidroside (Sal) mitigates hydrogen peroxide-induced injury by activating the PI3K/AKT and Wnt/b-catenin pathways by increasing miR-27a.	Zhao et al. (2019a)
miR-199-5p	Targets the 3’-UTR of TGF *β*_2_. The increase in TGF β_2_expression induced by oxidative stress may be related to the downregulation of mir-199-5p expression.	Feng (2014)
miR-182	MiR-182 expression is upregulated in primary TMCs with stress-induced premature senescence. The overexpression of miR-182 contributes to the phenotypic alterations of senescent cells.	Liu et al. (2016)
miR-183	Decreases the expression of laminin, gel, and type I collagen by targeting ITG β1 without a 3’-UTR.	Li et al. (2010)
miR-450	Influences the shrinkage of TMCs by targeting the MyoD family of proteins.	Sun et al. (2014)
miR-107	Regulates Nestin expression and counteracts the apoptosis of TMCs.	Xue et al. (2006)
miR-144-3p	The over-expression of miR-144-3p promotes the proliferation and invasion of TMCs by inhibiting the expression of FN-1 in oxidative stress TMCs	Yin & Chen (2019)
LncRNA-RP11-820	Promotes ECM production via regulating miR-3178/MYOD1	Shen et al. (2019)
LncRNA antisense noncoding RNA in the INK4 locus (ANRIL)	Down-regulates microRNA-7 to protect TMCs in an experimental model for glaucoma	Zhao et al. (2019b)

** (4) PUFAs**

Polyunsaturated fatty acids (PUFAs) have numerous anti-inflammatory and antioxidant properties (Sacca et al., 2018) that can influence mitochondrial energy production; improve mitochondrial function (Putti et al., 2015); influence cellular energy metabolism, neuronal plasticity, and membrane homeostasis (Dyall, 2017); and improve synaptic function. Omega-3 and omega-6 fatty acids exert preventative effects against oxidative stress in TMCs by abolishing the stimulation of NF-κB and IL-6. Therefore, the physiological basis of the PUFA-mediated protection of TMCs from oxidative stress has been revealed, which may provide new targets for antioxidation treatment (Tourtas et al., 2012).

** (5) Exogenous compounds**

Due to the association between oxidative stress and age-related disease, many types of phytochemicals, including polyphenols and terpenoids, which have anti-inflammatory and antioxidant properties, have been reported to be potential preventative treatments for ocular diseases. Additionally, other compounds, including rapamycin (He et al., 2019), ethyl pyruvate (Famili, Ammar & Kahook, 2013), and 1α,25-dihydroxyvitamin D3 (Lv et al., 2019), exert protective effects against oxidative stress through different pathways. The functions and mechanisms of these compounds are shown in Table 2. Studies investigating exogenous compounds have revealed new treatment options for oxidative stress.

**Table 2:** Role of antioxidative exogenous compounds.

Name	Functions and Mechanisms	References
Resveratrol	Increases mitochondrial mass and mitochondrial DNA. Activates SIRT1 and upregulates NO and eNOS. Activates Nrf_2_ pathways.	Avotri, Eatman & Russell-Randall (2019), Coralia et al. (2011)
Lycium barbarum polysaccharides (LBP)	Activates the PI3K/AKT and ERK signaling pathways by upregulating miR-4295.	Liu & Zhang (2019)
Curcumin	Inhibits proinflammatory factors, including IL-6, ELAM-1, IL-1α, and IL-8, decreases the activities of the senescence marker SA-β-gal, and lowers the levels of carbonylated proteins and the number of apoptotic cells.	Lin, Ciolino & Pasquale (2017)
Baicalin	Increases cell survival and decreases iROS production. Inhibits the production of IL-1α and ELAM-1, decreases the activity of senescence-associated SA-β-gal, and lowers the level of carbonylated proteins.	Gong & Zhu (2018)
Sulforaphane	Attenuates H_2_O_2_-induced oxidative stress via PI3K/AKT-mediated NRF2 signaling activation.	Liu & Zhang (2019)
Quercetin	Upregulates antioxidant peroxiredoxins through the activation of the NRF2/NRF1 transcription pathway and protects against oxidative stress-induced ocular disease.	Naoya et al. (2011)
Procyanidins	Decreases the apoptotic rate of TMCs under oxidative stress and reduces the release of cytochrome C.	Shi & Wang (2017)
Salidroside	Protects TMCs against H_2_O_2_-induced oxidative damage by activating the PI3K/AKT and Wnt/β-catenin pathways by increasing miR-27a.	Zhao et al. (2019a), Zhao et al. (2019b)
Polyphenols (derived from red wine, tea and dark chocolate)	Targets eNOS and induces the accumulation of NRF2.	Mann et al. (2007), Upadhyay & Dixit (2015)
Rapamycin	Protects TM-1 cells from COS by inhibiting mTOR and inducing autophagy. In addition, removes damaged mitochondria.	He et al. (2019)
Ethyl pyruvate	Able to nonenzymatically reduce hydrogen peroxide and scavenge hydroxyl radicals.	Dobsak et al. (1999), Famili, Ammar & Kahook (2013)
1α,25-dihydroxyvitamin D3	Attenuates OS-induced damage in TMCs by inhibiting TGFβ-SMAD3-VDR pathway	Lv et al. (2019)

**(6) Systemic antioxidant administration for glaucoma** treatment

As described in the section “TM oxidative stress and glaucoma”, systemic antioxidant capacity can reflect local ocular redox status. Some researchers have hypothesized and verified that increases in systemic antioxidant levels due to long-term antioxidant intake can increase local antioxidant levels, but the evidence is limited. Intake of vitamins C, A, and E is not significantly associated with the risk of POAG (Kang et al., 2003; Wang, Singh & Lin, 2013). Notably, in the case of glaucoma, systemic drugs have greater difficulty crossing the blood-retinal barrier than local drugs (Lin, Ciolino & Pasquale, 2017); in addition, systemic drugs have more systemic side effects and lower bioavailability than local drugs. These differences remain challenges to be solved. Many new drug delivery systems (such as in situ gels, liposomes, niosomes, hydrogels, dendrimers, nanoparticles, and solid lipid nanoparticles) are in clinical trials. The goal of related research is to improve drug delivery in appropriate recipients, which may improve efficacy and compliance and reduce side effects (Yadav, Rajpurohit & Sharma, 2019).

## Conclusion

Various studies on humans and laboratory animals have demonstrated that a variety of antioxidants, particularly noncoding RNAs and exogenous compounds, help to regulate IOP and protect TMCs from oxidative stress. Based on these studies, it is believed that new methods with broad applicability and promise for the treatment of oxidative stress and glaucoma will be developed in the near future.

## Additional Information and Declarations

### Data Availability

The following information was supplied regarding data availability: This is a literature review.
